# Symmetrical Drug-Related Intertriginous and Flexural Exanthema With Amoxicillin: Presenting a Flare-Up Phenomenon

**DOI:** 10.7759/cureus.74393

**Published:** 2024-11-25

**Authors:** Patricia Letón-Cabanillas, Alejandra Jover-Walsh, Gabriela Aray, Blanca Noguerado-Mellado, Patricia Rojas Perez-Ezquerra

**Affiliations:** 1 Allergy Department, Hospital General Universitario Gregorio Marañón, Madrid, ESP

**Keywords:** amoxicillin, beta-lactams, delayed hypersensitivity, drug allergy, flare-up, intradermal skin test, penicillin

## Abstract

This case report describes a 40-year-old male patient who developed symmetrical drug-related intertriginous and flexural exanthema after taking amoxicillin. Initial allergy testing showed negative intradermal tests, but subsequent drug provocation tests with amoxicillin and penicillin were positive, indicating cross-reactivity between these β-lactam antibiotics. Notably, following the final provocation test, the intradermal test with penicillin turned positive, demonstrating a flare-up phenomenon. The findings highlight the importance of thorough allergy evaluations, including drug provocation testing, to guide safe antibiotic treatment.

## Introduction

Symmetrical drug-related intertriginous and flexural exanthema (SDRIFE) is an infrequent cutaneous adverse reaction characterized by symmetrical reddening and swelling in body folds and flexural areas, typically occurring after exposure to certain medications [1]. Intertriginous areas refer to parts of the body where skin touches or rubs together, such as the armpits, groin, and under the breasts. SDRIFE has been reported with various medications, most commonly antibiotics like amoxicillin and other beta-lactams (BLs) [1-3]. Patients with SDRIFE typically develop a symmetrical, well-demarcated rash in flexural areas within hours to days after drug exposure, often without other systemic symptoms [1-3]. SDRIFE is classified as a type IV hypersensitivity reaction defined by five criteria: exposure to a systemically administered drug, sharply demarcated erythema in specific areas, involvement of additional intertriginous/flexural locations, symmetry of affected regions, and absence of systemic symptoms [2]. While SDRIFE is uncommon, recognizing it is important for proper diagnosis and management of drug reactions.

This case report not only presents a typical SDRIFE reaction but also describes a rare flare-up phenomenon during allergy testing. The flare-up phenomenon consists of a shift from negative to positive skin test (ST), normally an intradermal test (IDT), after systemic exposure to the drug used for ST [4]. We present a case of SDRIFE induced by amoxicillin, featuring a flare-up phenomenon on an ST following a drug provocation test (DPT) with penicillin.

This article, with variations, was previously posted to the Research Square preprint server on May 01, 2024.

## Case presentation

A 40-year-old male patient underwent dental extraction, for which amoxicillin was prescribed as prophylaxis, having been previously well-tolerated. Four days after initiating the antibiotic, the patient developed papular erythematous lesions forming desquamative plaques with mild infiltration bilaterally in the gluteal, armpit, and groin areas. There was no mucosal involvement or systemic symptoms. Amoxicillin was discontinued and rotated to clindamycin, which was well-tolerated, and oral prednisone and oral ebastine were initiated, leading to the complete resolution of the exanthema within 15 days without any residual lesions.

**Figure 1:**
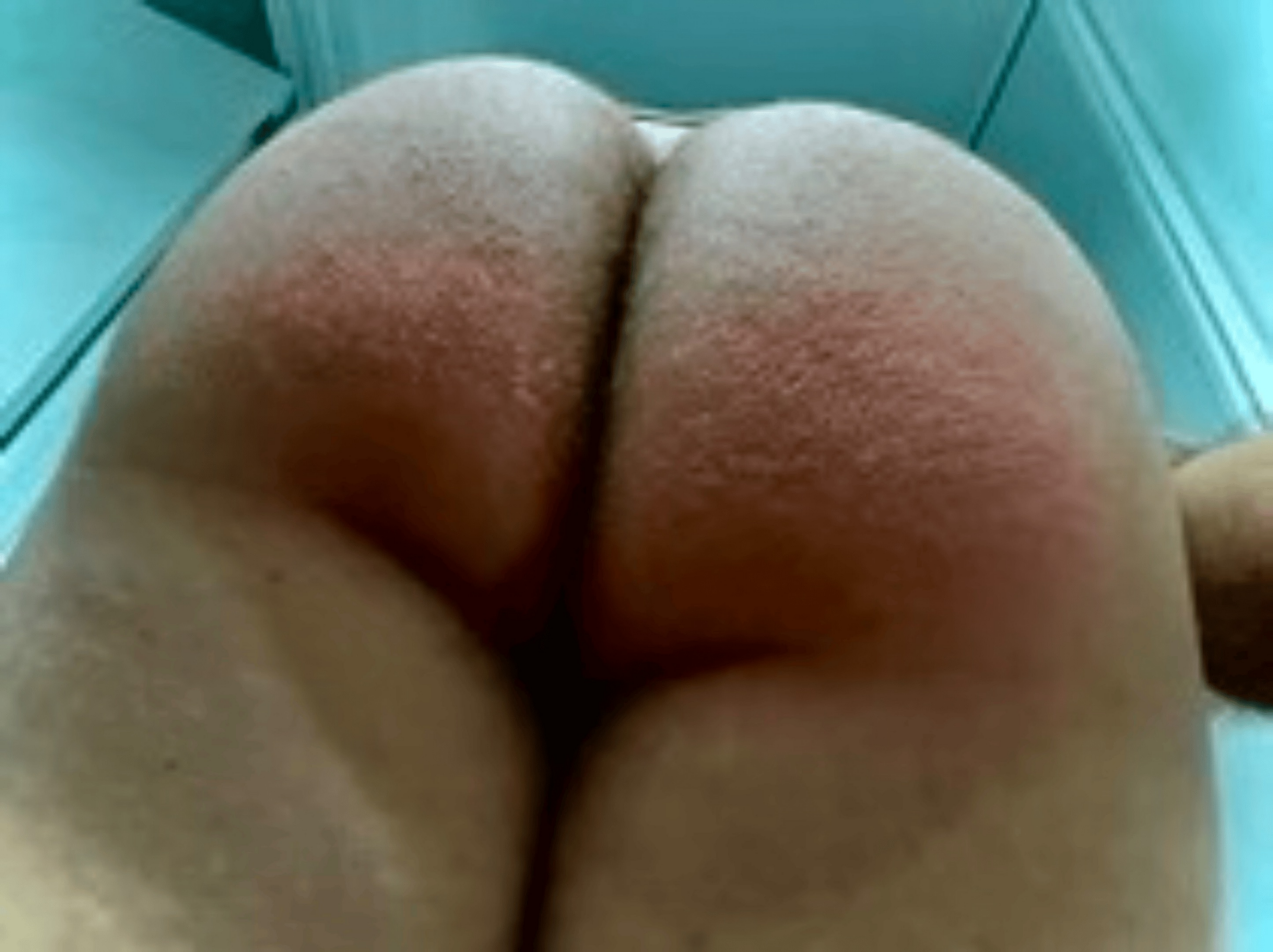
Bilateral gluteal erythematous plaques following positive amoxicillin drug provocation test

Five months later, after signed written consent was obtained, an allergy workup was conducted. In the first place, IDT with benzylpenicilloyl polylysine (BPO) extract (6 x 10-5 M), minor-determinant mixture (MDM) extract (0.5 mg/mL), freshly prepared penicillin G (10.000 units/mL), and amoxicillin extract (20 mg/mL) (Lab Diater®, Madrid, Spain), was performed with negative readings at 15 minutes and 24 hours. Therefore, a DPT with amoxicillin was initiated following our protocol for delayed reactions. On the first day, half of the minimum effective dose (250 mg) was administered. The following day, the full dose (500 mg) was given. Subsequently, the patient continued with home treatment at a dosage of 500 mg every 12 hours. After three days of home treatment, the patient experienced bilateral pruritic and erythematous plaques in the gluteal and inguinal regions (Figure 1).

**Figure 2:**
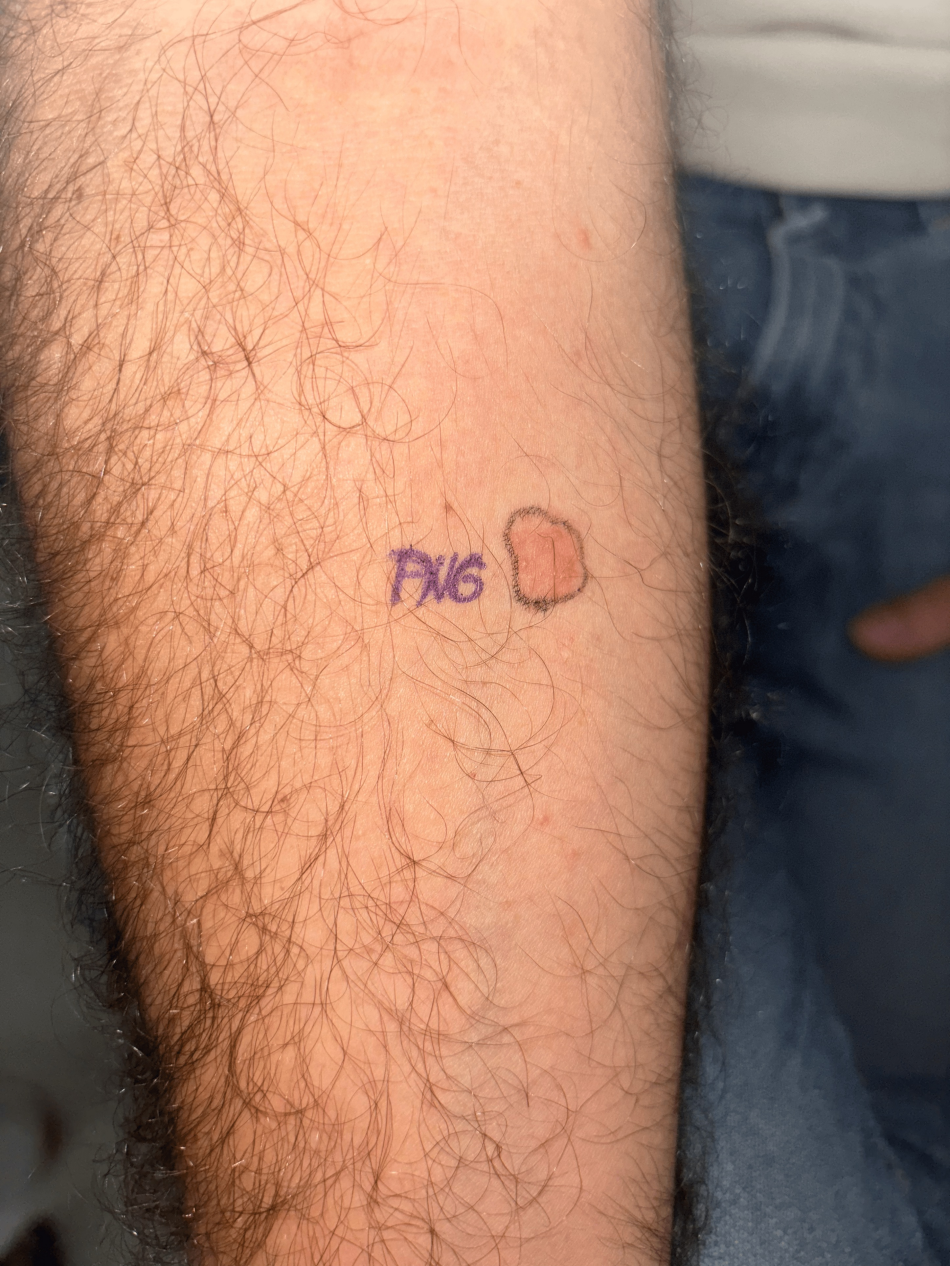
Flare-up phenomenon Penicillin intradermal test (IDT) shifts from negative to positive, showing an infiltrated erythematous macula after systemic administration

STs were performed again to study cross-reactivity with other BL antibiotics. IDTs with BPO, MDM, penicillin G, and amoxicillin were negative in readings at 15 minutes and 24 hours. Consequently, one week later, a DPT with penicillin was performed. With a single dose of 500 mg, 12 hours after the intake, the patient presented again with bilateral pruritic and erythematous plaques in the gluteal and inguinal regions, and at the same time, the IDT with penicillin showed an infiltrated erythematous macula (Figure 2). This phenomenon is known as a flare-up.

The patient was finally diagnosed with SDRIFE induced by amoxicillin with cross-reactivity due to sensitization to the BL ring. As a result of this diagnosis, avoidance of all BL antibiotics was recommended; since then, he has not experienced any further reactions.

## Discussion

The most notable aspect of this case report is the observed flare-up phenomenon following penicillin DPT. This phenomenon, characterized by a shift from negative to positive ST results after systemic exposure to a drug, is typically described in delayed reactions. In this instance, the IDT with penicillin, initially negative, became positive following DPT with the same drug. This phenomenon has not been documented before with penicillin. The underlying mechanism of the flare-up phenomenon is not fully understood. However, it is hypothesized that skin-resident memory T cells (CD4+ CCR10+) may play a crucial role in reactivating the immune response upon re-exposure to the offending drug [4]. This case adds to the limited literature on flare-up reactions. It has been described with IDTs and patch tests mainly; there is one rare case after a prick test [5]; and with amoxicillin [4], ampicillin, BPO and MDM [6,7], paracetamol [8], trimethoprim-sulfamethoxazole [9], ibuprofen [5], heparin [10], and glatiramer acetate [11].

Another key finding is the cross-reactivity between amoxicillin and penicillin, evidenced by positive DPTs with both antibiotics. This result strongly suggests sensitization to the shared BL ring structure, a crucial consideration in managing patients with suspected BL allergies [12].

As a secondary point, this case also presents a typical manifestation of SDRIFE induced by BL antibiotics. SDRIFE, first described in 1984 as "baboon syndrome" with mercury [13], is a rare cutaneous adverse reaction characterized by symmetrical erythema and edema in intertriginous and flexural regions, often associated with medication exposure. The management of SDRIFE typically involves discontinuation of the offending drug and the initiation of corticosteroids and antihistamines, as was done in this case. The complete resolution of the exanthema within 15 days without residual lesions highlights the effectiveness of this approach [1].

This case report contributes to the growing body of literature on SDRIFE. While SDRIFE has been previously reported with BL antibiotics, this case is unique in demonstrating a flare-up reaction after positive DPT with penicillin. 

## Conclusions

In summary, we present a documented case of a flare-up phenomenon with penicillin and SDRIFE induced by amoxicillin. This was confirmed through positive DPTs with both amoxicillin and penicillin, indicating cross-reactivity due to sensitization to the BL ring. Notably, the patient experienced a flare-up phenomenon at the site of the initial IDT with penicillin after a positive DPT with that drug.

This clinical case is significant as it illustrates a phenomenon rarely encountered in routine clinical practice. Allergists should be aware and learn to recognize this phenomenon to ensure accurate diagnosis and appropriate management of drug allergies. The case provides valuable insights into the complex nature of drug hypersensitivity reactions, particularly the flare-up phenomenon with penicillin. Future research directions could include further investigation into the mechanisms underlying the flare-up phenomenon, particularly the role of skin-resident memory T cells. This case serves as a reminder for clinicians to remain vigilant for unusual manifestations of drug allergies and highlights the need for continued research in this field.
